# Editorial: Diagnosis and management of acute, chronic, and autoimmune pancreatitis

**DOI:** 10.3389/fmed.2024.1517007

**Published:** 2024-11-14

**Authors:** Ravi Kumar Sharma, Francesco Vitali, Puneet Chhabra

**Affiliations:** ^1^Department of University Institute of Biotechnology, Chandigarh University, Mohali, Punjab, India; ^2^Department of Internal Medicine, A University Hospital of Greifswald, Greifswald, Germany; ^3^Calderdale Royal Hospital, Halifax, United Kingdom

**Keywords:** acute pancreatitis, chronic pancreatitis, autoimmune pancreatitis, biomarkers and diagnosis, management, editorial

Pancreatitis represents an inflammatory disease of the pancreatic gland with multiple genetic and environmental etiological factors that cause considerable clinical concern commonly associated with hospital admissions, increased morbidity, and mortality worldwide (1). According to the clinical course, pancreatitis is classified as acute and chronic, although sometimes an overlap between the two entities, like in recurrent pancreatitis, can be found (2). A progression from acute to chronic pancreatitis has also been described (3). Autoimmune pancreatitis (AIP) is a rare disease with a low annual incidence, which varies substantially between geographical regions (4, 5).

AP is defined by sudden pancreatic inflammation, involving the acini and the pancreatic ducts. It's an unpredictable and potentially fatal illness. The prognosis is primarily determined by the development of organ failure, pancreatic or peripancreatic necrosis and subsequent infection. Gallstones, alcohol usage, hyperlipidaemia, or certain drugs are the most common causes. Severe abdominal pain (often radiating to the back), high serum amylase and lipase levels, and signs of pancreatic edema or inflammation on imaging techniques such as abdominal ultrasonography or CT scans are common features (6).

AP is managed primarily by supportive treatment, which includes fluid resuscitation, pain management, and nutritional assistance. Severe cases may require radiological and endoscopic intervention in a step-up approach manner, especially if patients develop complications such as abscess or suprainfection of necrosis (7, 8).

CP results from fibro-inflammatory changes of the pancreatic gland, leading to irreversible damage and functional impairment. Etiologies often include long-term alcohol abuse, tobacco smoking, genetic factors, or metabolic disorders (9). Clinically, patients may present with abdominal pain, malabsorption, and diabetes. The diagnosis is established through a combination of history, imaging, and functional tests assessing pancreatic enzyme secretion. Management emphasizes pain relief, lifestyle modification and enzyme replacement therapy. In some cases, surgical options, such as ductal decompression or resection, may be indicated. CP patients have a lifetime risk of developing pancreatic ductal adenocarcinoma (10).

AIP, an entity often classified as a chronic pancreatitis, is characterized clinically by obstructive jaundice with or without a pancreatic mass. It is often associated with other autoimmune conditions. It is categorized into two types: Type 1, which is associated with IgG4-related disease, and Type 2, which is associated with inflammatory bowel disease and histologically characterized by granulocytic ductal infiltrations (GEL: granulocytic epithelial lesions)1 (11). Diagnosis relies on serological markers, imaging findings, and histological confirmation through biopsy. Management typically involves corticosteroids, which can lead to significant improvement. Long-term follow-up is critical, as relapse can occur (4, 5).

The current Research Topic on pancreatitis encompasses a wide range of significant contributions in the form of original articles. Yang et al. demonstrated that modulation of the PI3K/AKT signaling pathway can enhance apoptosis in pancreatic acinar cells while simultaneously reducing the inflammatory response, highlighting a potential therapeutic approach. Similarly, Li et al. identified smoking as an independent risk factor, showing that it can significantly increase the severity of pancreatitis. Xing et al. developed a nomogram that performed well in predicting persistent organ failure (POF) in patients with acute pancreatitis (AP). This tool can support clinical decision-making and improve personalized treatment strategies. Ma et al. explored the causal associations between specific lipidome types and pancreatitis, contributing to a deeper understanding of lipid metabolism in the disease, which may pave the way for more targeted interventions. Lin et al. used Mendelian randomization analysis to suggest that there is no causal association between glucocorticoid use and the risk of pancreatitis, which helps clarify conflicting data on this therapeutic approach.

Wiese et al. highlighted that malnourished patients with chronic pancreatitis can significantly benefit from intensified nutritional therapy. In addition to improving nutritional status, a multimodal intervention can enhance muscle function and improve overall disease prognosis. Frost et al. showed that the composition of the stent microbiome is associated with prolonged hospital stays and adverse events during endoscopic drainage therapy. This finding underscores the importance of infection control to optimize patient outcomes during such procedures. Jia et al. demonstrated that the combination of traditional Chinese medicine and modern medicine in treating patients with mild to moderate acute pancreatitis effectively reduces inflammatory indicators and shortens both symptom duration and hospitalization periods, ultimately promoting faster disease recovery. Jia et al. demonstrated that integrating traditional Chinese medicine with modern medical treatments significantly reduces inflammatory markers in patients with mild to moderate AP. This approach helps shorten the duration of symptoms, reduces hospital stay, and accelerates recovery. Zahariev et al. demonstrated that factors such as the severity and recurrence of AP, along with etiologies like alcohol consumption and hypertriglyceridemia, as well as conditions like organ failure, pancreatic necrosis, obesity, chronic kidney and liver diseases, and dyslipidemia, are linked to an increased risk of developing prediabetes or diabetes.

Research continues to evolve, focusing on understanding the underlying mechanisms of pancreatitis, identifying biomarkers for early diagnosis, and developing targeted therapies. Enhanced collaboration between gastroenterologists, radiologists, and pathologists is essential to improve patient outcomes.

In conclusion, a comprehensive understanding of the diverse forms of pancreatitis is crucial for clinicians. By advancing diagnostic methods and tailoring management strategies, we can significantly impact patient care, mitigate complications, and improve overall quality of life for those affected by this complex condition.
